# P042. Mechanism of action and clinical evidence of botulinum toxin in chronic migraine

**DOI:** 10.1186/1129-2377-16-S1-A138

**Published:** 2015-09-28

**Authors:** Giovanni Franco, Eleonora Vecchio, Marianna Delussi, Katia Ricci, Anna Montemurno, Marina de Tommaso

**Affiliations:** Neurophysiopathology of Pain Unit, Neuroscience and Sensory Systems Department, University of Bari “Aldo Moro”, Bari, Italy

Considerable evidence exists supporting the notion that botulinum toxin type A (BoNT/A) can exert a direct analgesic effect in addition to its myorelaxant effect. It is likely that the benefict of using BoNT/A as prophylactic treatment for chronic migraine is due to its ability to inhibit overactivity of motor neurons and hyperexcitability of sensory neurons, by involving the suppression of peripheral and central sensitization.

In this study we aimed to evaluate the effects of BoNT/A on amplitude, latency and habituation of laser evoked potentials (LEPs) in patients with chronic migraine.

We recruited 20 patients with a diagnosis of chronic migraine treated with type A botulinum toxin every 3 months. LEPs were recorded in basal, two hours and ten days after both BoNT/A and placebo injection. Headache frequency, allodynia and total tenderness score (TTS) were evaluated at basal condition and after one-year of treatment.

We found N2 and P2 latency increased 10 days after toxin injection, while LEPs amplitude was not modified. Compared to placebo injection, the habituation of N2/P2 LEPs component obtained by stimulating supraorbital zone was significantly increased after BoNT/A infiltration. After ten days, habituation pattern in migraine patients was similar to that of normal subjects. In the one-year follow-up we observed a significant migraine frequency and allodynia improvement, but no effect on total tenderness score. Furthermore the habituation change correlated with the clinical effectiveness.

Study results suggest a toxin modulating action on nociceptive afferents in patients with chronic migraine. The N2 and P2 latency increase obtained from hand laser stimulation suggests a possible systemic pain inhibiting effect. Although the botulinum toxin did not show an inhibitory effect on trigeminal nociceptive system, it seems to improve the reduced habituation pattern which promotes the central sensitization. The therapeutic effect of BoTN/A seemed to be related to the effect on trigeminal habituation obtained after 10 days from the first infiltration, which could be considered as a potential neurophysiological pattern to predict the non-responders.

Results of this study confirm that the effect of botulinum toxin on chronic migraine may be related to a modulation and normalization of central sensitization mechanisms. The lack of effects on pericranial muscle tension precludes to suppose a modulating effect on trigeminal nociception by the inhibition of the neuromuscular synapse.

Written informed consent to publish was obtained from the patient(s).

